# Leaf Carbon Exchange of Two Dominant Plant Species Impacted by Water and Nitrogen Application in a Semi-Arid Temperate Steppe

**DOI:** 10.3389/fpls.2022.736009

**Published:** 2022-05-02

**Authors:** Xiaolin Zhang, Penghui Zhai, Jianhui Huang

**Affiliations:** ^1^College of Grassland Science, Shanxi Agricultural University, Taigu, China; ^2^State Key Laboratory of Vegetation and Environmental Change, Institute of Botany, The Chinese Academy of Sciences, Beijing, China; ^3^College of Resources and Environment, University of Chinese Academy of Sciences, Beijing, China

**Keywords:** light response curve, nitrogen application, photosynthesis, snow addition, water addition, *Leymus chinensis*, *Stipa grandis*

## Abstract

Photosynthetic characteristics are widely used as indicators of plant responses to global environmental changes such as precipitation change and nitrogen (N) deposition increase. How different plant species respond physiologically to the future precipitation change combined with increasing N availability is largely unclear. A field experiment was conducted to study responses in seasonal and interannual leaf carbon (C) exchange of two dominant plant species, *Leymus chinensis* and *Stipa grandis*, to additional water (either as spring snow or as summer water) and N application in a semi-arid temperate steppe of China. Our results showed that spring snow and summer water addition both increased the maximum photosynthetic rate (A_max_) of two dominant species. Such effect was likely caused by raised light saturation point, the maximum apparent quantum yield, stomatal conductance, and transpiration rate. The N application combined with spring snow or summer water addition both enhanced A_max_ of *S. grandis* in both experimental years, whereas N application only increased A_max_ of *L. chinensis* combined with summer water addition. Their responses were attributed to a concurrent increase in leaf N concentration (N_leaf_) and decrease in leaf phosphorus (P) concentration (P_leaf_), indicating that N_leaf_ and P_leaf_ affect photosynthetic characteristics to regulate leaf C exchange. Our results suggest that differentiated responses among different species in photosynthetic characteristics may lead to changes in ecosystem structure and functioning under increasing precipitation and N deposition.

## Introduction

Ecosystem processes have been strongly modified by global change factors in terms of changes in both patterns and amount of precipitation and an increase in the amount of atmospheric nitrogen (N) deposition. Water and N are the two most limiting factors for plant growth and carbon (C) sequestration at both the leaf and ecosystem levels (Liang et al., 2020; Zhang et al., 2021). The two factors can also interact in intricate ways to influence the above-mentioned processes (Tang et al., 2017). By the end of the twenty-first century, rainfall is predicted to significantly increase in China with an increase of up to 30% in northern region (Cholaw et al., 2003; Chen, 2013) and snowfall is also predicted to greatly increase in the near future (Ma et al., 2012). Generally, rainfall is far more important than the snowfall in terms of their relative amount in the total precipitation in Inner Mongolia (Zhang et al., 2015). In addition, changes in precipitation amount will inevitably influence soil water content and nutrient availability, resulting in alteration in photosynthesis, and consequently affect plant growth (Davis et al., 1999; Ashraf and Harris, 2013). Besides, increasing N deposition due to industrialization globally and intensified use of N fertilizers, especially in agricultural systems will finally affect biogeochemical processes by changing soil N availability and other soil physicochemical properties (Galloway et al., 2008). Specifically, N deposition could induce water stress suppressing stomatal conductance and transpiration and further influence leaf C exchange (Zhang B. et al., 2017).

Leaf photosynthetic characteristics including photosynthetic capacity (i.e., the maximum photosynthetic rate, A_max_), stomatal conductance, and transpiration rate can reflect plant responses to environmental changes and play fundamental roles in plant growth, which can further impact ecosystem structure and functioning (Xu et al., 2013). Plant chemistry, such as leaf N concentration (N_leaf_) and leaf phosphorus (P) concentration (P_leaf_), can be regarded as critical indicators for the response of different plants to those global change factors such as increasing N deposition and precipitation alteration (Reich, 2005). In addition, N_leaf_ and P_leaf_ are tightly correlated with the photosynthetic rate because they are important constituents for the synthesis of metabolic enzymes, chlorophyll, and other relevant photosynthetic apparatus (Gao et al., 2018; Zhong et al., 2018). Previous studies have shown that there are generally positive effects of N addition on plant photosynthesis (Zhang et al., 2008; Hou et al., 2019; Liang et al., 2020), although insignificant or even negative effects are also reported (Reich et al., 2003; Lin et al., 2017; Zhai et al., 2017). Therefore, it is highly needed to study the effects of N application on the seasonal and interannual variation in photosynthetic characteristics of different plant species, especially when combined with changes in water supply.

Dominant species generally determine community structure, function, and dynamics (Orwin et al., 2014; Mason et al., 2016), and they may have a broader range of physiology and therefore play a damping role on ecosystem fluctuation under future global changes (Norberg et al., 2001; Jump and Penuelas, 2005). In Inner Mongolia grasslands, *Leymus chinensis* and *Stipa grandis* are two dominant plant species in the typical steppe. They have distinct morphological and physiological traits with *L. chinensis* requiring relatively wetter and more fertile habitats than *S. grandis* (Chen et al., 2005). Therefore, the two species may respond differently to changes in water and N supply. Although the two species have established a stable balance by competing for limited resources when they co-exist in a community, changes in precipitation and N deposition may disturb and break this balance by affecting their relative competitiveness. Changes in their competitiveness inevitably impact their distribution, then reshape the community by shift in species composition, and, consequently, affect C sequestration and other processes at the ecosystem level (Walther et al., 2002; Still et al., 2003; Niu et al., 2006). Our previous study found that *L. chinensis* showed stronger responses than *S. grandis* in terms of abundance and biomass production under increasing precipitation and enhanced N availability in the semi-arid steppe (Zhang et al., 2021). However, little is known about the underlying mechanisms such as their responses in leaf photosynthetic characteristics of the two species under future scenarios of precipitation change and N deposition increase.

In this study, we would like to examine how plants, especially for the two dominant species (*L. chinensis* and *S. grandis*) in a typical steppe, respond to changes in precipitation regime either by increasing spring snow or by increasing summer rainfall interacted with N addition in terms of leaf C exchange capacity based on the construction of light response curves. We also aimed to understand what were the underlying mechanisms that would induce these responses. We hypothesized that (1) spring snow addition and summer water addition would have significantly different effects on leaf C exchange of the two species; (2) N addition would affect the leaf C exchange of the two species differently under spring snow and summer water addition; and (3) *L. chinensis* would respond stronger than *S. grandis* in the N uptake, photosynthetic characteristics, and would thus shift community structure and further influence ecosystem functioning under future global change scenarios.

## Materials and Methods

### Site Description

This study was conducted in a typical steppe located near the Inner Mongolia Grassland Ecosystem Research Station, Institute of Botany, Chinese Academy of Sciences (43°33′N and 116°40′E; 1,251 m above sea level). The studied typical steppe is primarily dominated by two grass species, namely *Leymus chinensis* and *Stipa grandis*. The regional monsoon climate in the study area is characterized by cold, dry winters and warm, wet summers. The long-term mean annual temperature (1970–2013) is 0.4°C (ranging from −24.1°C in January to 19.6°C in July). The mean annual precipitation is 333.3 mm (1982–2013), and the majority of precipitation occurs in the form of rainfall (91.9%, ~280.5 mm from May to September) with relatively small snowfall (8.1%) (Zhang et al., 2021).

### Experimental Design and Treatments

A randomized block design was used in the experiment with treatments of N and water application. For this study, there were three replicates which were set up in the site and six treatments were involved. Therefore, totally 18 plots (5 m × 5 m) were included in this study with at least 1-m distance between adjacent plots. The six treatments were as follows: no water supply and no N application (control; N0W0), spring snow addition (N0W1), summer water addition (N0W2), N application (N1W0), spring snow and N application (N1W1), and summer water and N application (N1W2). Approximately 25 mm water equivalent of snow from the nearby area was evenly added to each spring snow plot in early March. Totally, 100 mm water from the nearby river was added to each summer rainfall plot yearly with a handheld sprinkler irrigation system, starting on June 15, 2010 (10 mm weekly). For N application, 10 g N m^−2^ was added to the plots in the form of urea in early July each year starting in 2009. This study was conducted for two growing seasons from 2012 to 2013.

### Leaf C Exchange

Either the first or the second fully extended and intact leaf of each studied plant species was selected from each plot for leaf C exchange measurement. Measurements were taken between 08:00 am and 11:30 am once every 2 weeks from the end of May to the end of September with nine-time measurements across the years 2012 and 2013. Net photosynthetic rate (P_n_), stomatal conductance, and transpiration rate were recorded using a LI-6400 portable photosynthesis system (Li-Cor Inc., Lincoln, NE, USA) with a red–blue LED source. Light response curves were obtained based upon P_n_ measurements along changes of photosynthetically active radiation (PAR) from 0 to 2,000 μmol m^−2^ s^−1^ (with the sequence of 2,000, 1,500, 1,200, 1,000, 800, 500, 300, 200, 150, 100, 50, 20, and 0 μmol m^−2^ s^−1^) using the built-in light source in the natural condition. Before the measurements, leaf was adapted to light under 2,000 μmol m^−2^ s^−1^ and then the data were logged in turn when the parameters were stable with ΔCO_2_ value fluctuation of <0.2 μmol mol^−1^ and the P_n_ value was stable at one after the decimal point. By fitting data to a quadratic equation (Equation 1), we determined the following physiological parameters of the two target plants, including the maximum photosynthetic rate (A_max_) representing plant photosynthetic capacity, light saturation point representing the value of PAR when photosynthesis maintains relative high level without further change, light compensation point representing photosynthesis that will be higher than respiration beyond this value of PAR, the maximum apparent quantum yield representing the efficiency of light utilization in photosynthesis, and respiration (Long et al., 1993).


(1) $$\begin{array}{r} {\text{P}}_{\text{n}}=\frac{\text{AQ}{\text{E}}^{*}\text{PAR}+{\text{A}}_{\max}-\sqrt{{\left(\text{AQ}{\text{E}}^{*}\text{PAR}+A_{\max}\right)}^{2}-4{\text{k}}^{*}\text{AQ}{\text{E}}^{*}\text{PA}{\text{R}}^{*}A_{\max}}}{2k}-R \end{array}$$

where P_n_ is the net photosynthetic rate (μmol CO_2_ m^−2^ s^−1^), A_max_ is the maximum photosynthetic rate (μmol CO_2_ m^−2^ s^−1^), AQE is the maximum apparent quantum yield of CO_2_ (μmol CO_2_ mol^−1^ photons), PAR is the photosynthetically active radiation (μmol m^−2^ s^−1^), *k* is the convexity coefficient (0 < *k* <1), and *R* is the rate of dark respiration (μmol CO_2_ m^−2^ s^−1^).

### Soil Temperature and Soil Moisture

Soil temperature at 5-cm depth was recorded using the TH-212 portable intelligent data logger (Hichance Inc., Haidian, Beijing, China) along with the photosynthetic measurement. Simultaneously, soil moisture to 10-cm depth was determined using a TDR-200 probe (Spectrum Technologies Inc., Plainfield, IL, USA).

### Leaf N and P Concentrations

To determine leaf N and P concentrations (N_leaf_ and P_leaf_), fresh leaves of *L. chinensis* and *S. grandis* were collected during peak plant growth in August 2012 and 2013, oven-dried at 65°C for ~48 h, and then ground to a fine powder using a ball mill (Retsch MM 400; Retsch, Haan, Germany). The N_leaf_ was determined using an Elemental Analyzer Vario Macro Cube model with the dry combustion method (Elementar Analysensysteme GmbH, Germany). The P_leaf_ was determined using the molybdenum blue colorimetric method at 880 nm after digesting the samples in a mixture of concentrated sulfuric acid and hydrogen peroxide (Tan et al., 2013).

### Statistical Analysis

Linear mixed models were used to determine the main effects of water, and N application and their interactive effects on the two dominant species (*L. chinensis* and *S. grandis*) in both growing seasons, separately. All response variables were checked for normality and homogeneity of model residues, and log10 or sqrt transformation was used if necessary.

We analyzed spring snow with or without N application treatments (N0W0, N0W1, N1W0, and N1W1) and summer water with or without N application treatments (N0W0, N0W2, N1W0, and N1W2) as different treatment levels, respectively. In the model, water including spring snow or summer water addition, and N application were treated as fixed terms. The sampling date was treated as a random term. The A_max_, light saturation point, light compensation point, the maximum apparent quantum yield, respiration, stomatal conductance, and transpiration rate were treated as response variables. The response variable model was selected by comparing several models according to maximum-likelihood estimation with the lowest AIC value (West, 2009). The final model estimates reported were based on restricted maximum-likelihood estimates.

Two-way analysis of variance (ANOVA) was used to determine the main effects of various treatments on N_leaf_ and P_leaf_. Student's *t*-tests were used to compare the differences between spring snow and summer water addition and the differences in N application between spring snow and summer water addition. The relationships of A_max_ with soil moisture, soil temperature, and precipitation (accumulated from previous November to October amount) were explored using curve fitting regression analysis. All data were analyzed using SPSS 23.0 for Windows (SPSS Inc., Chicago, IL, USA).

## Results

### Light Response Curves

**Figure 1:**
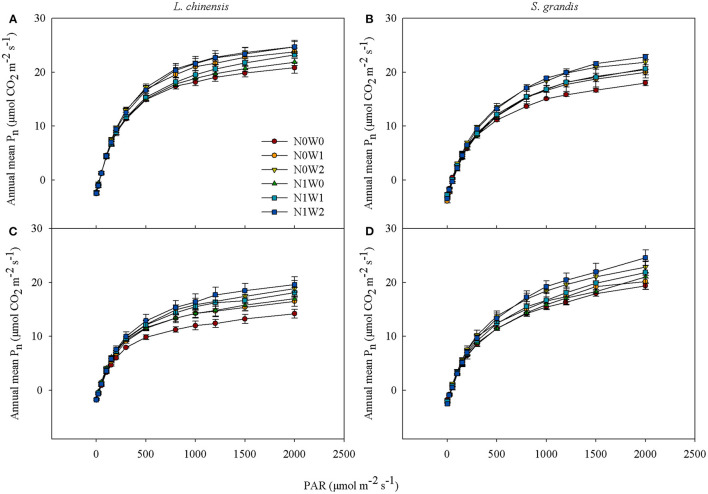
Average values of light response curves of *Leymus chinensis* (left panels) and *Stipa grandis* (right panels) across growing seasons in 2012 **(A,B)** and 2013 **(C,D)**, respectively. Values represent mean ± SE (*n* = 3). Treatments are designated as follows: N0W0, no water supply and no N application (control); N0W1, spring snow addition; N0W2, summer water addition; N1W0, N application; N1W1, spring snow and N application; and N1W2, summer water and N application.

By pooling data of measurements in each growing season, the average net photosynthetic rate (P_n_) of the two species, *L. chinensis* and *S. grandis*, all showed a saturating response pattern to change of PAR, that is, increased rapidly with PAR when PAR was relatively low, but leveled off when PAR reached a saturation point regardless of treatments in the two growing seasons (Figure 1). The slope of light response curves of both plant species showed no differences when PAR was at the light limitation stage (≤ 200 μmol m^−2^ s^−1^). However, P_n_ of the two species differentiated when PAR was at the light saturation stage with summer water addition showing greater increasing effects than spring snow addition with or without N addition. Besides, the annual average P_n_ of *L. chinensis* was greater than that of *S. grandis* in 2012 (*P* < 0.001), but turns opposite in 2013 when the value of PAR was > 800 μmol m^−2^ s^−1^ (*P* < 0.01).

**Figure 2:**
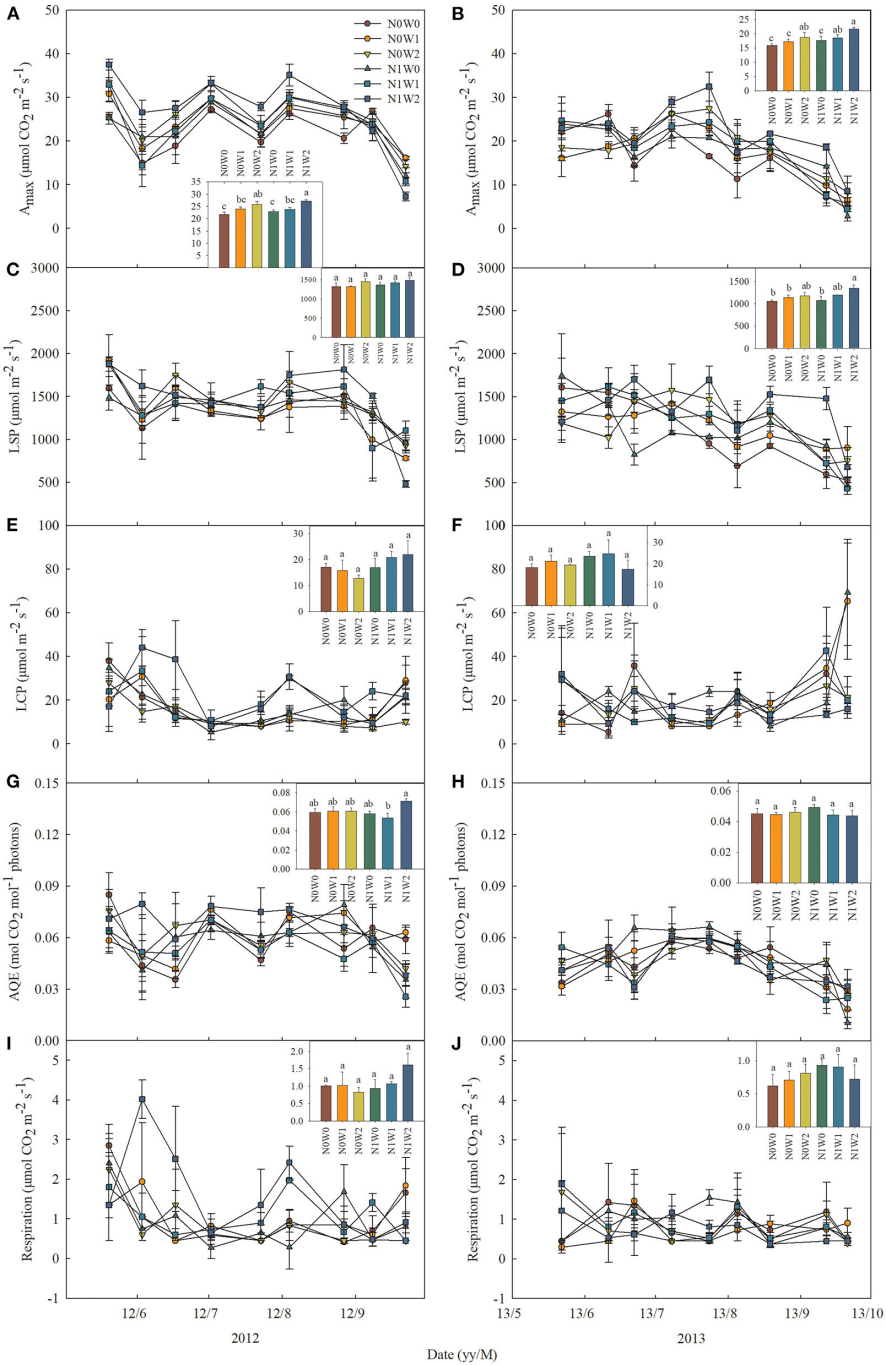
Seasonal dynamics of leaf photosynthetic characteristics of *Leymus chinensis* in 2012 (left panels) and 2013 (right panels), respectively, including **(A,B)** the maximum photosynthetic rate (A_max_), **(C,D)** light saturation point (LSP), **(E,F)** light compensation point (LCP), **(G,H)** maximum apparent quantum yield (AQE), and **(I,J)** respiration. Inset figures show the average values across seasons with different letters indicating significant differences (*P* < 0.05) among the six treatments. Values represent mean ± SE (*n* = 3).

**Figure 3:**
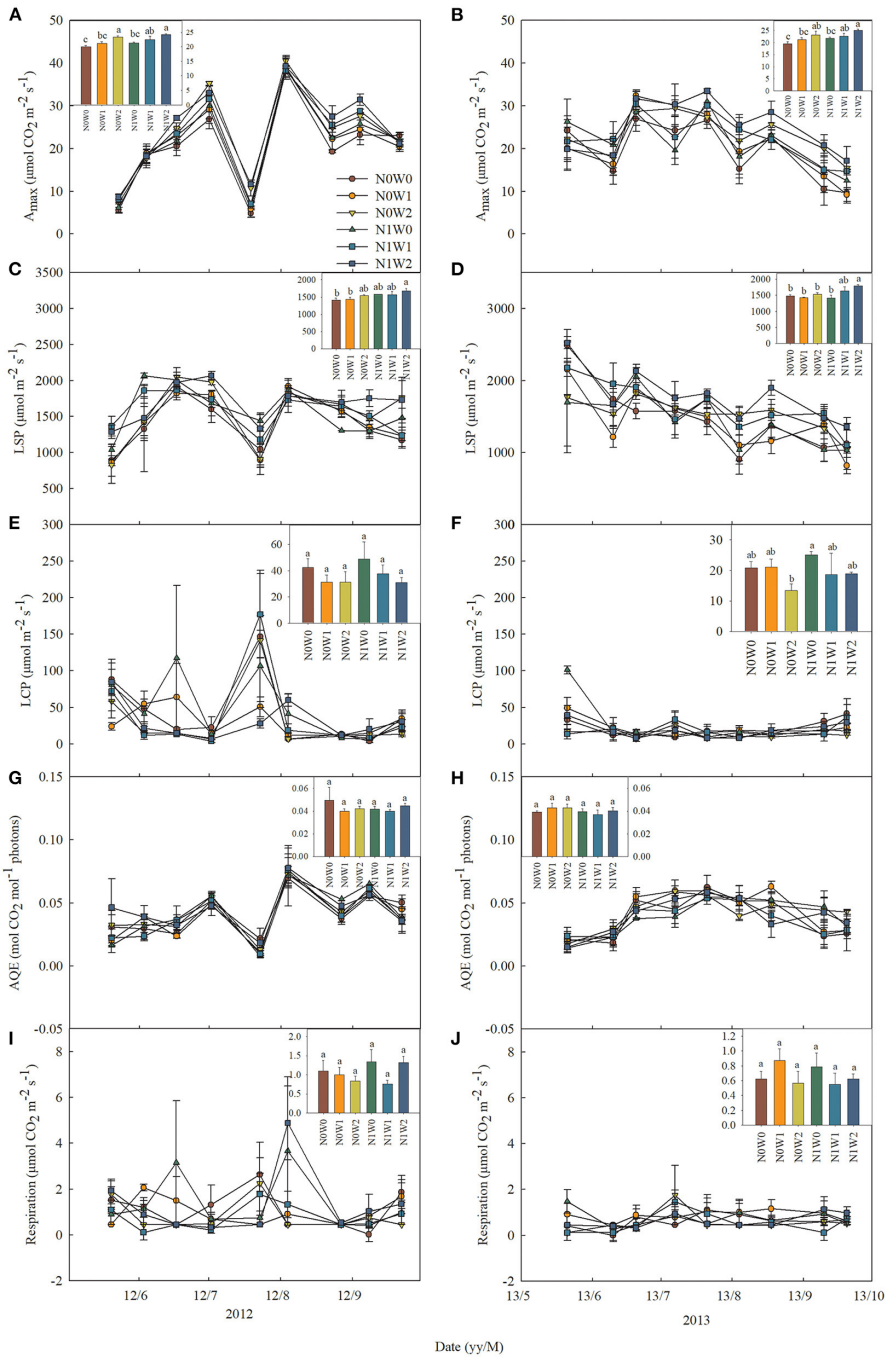
Seasonal dynamics of leaf photosynthetic characteristics of *Stipa grandis* in 2012 (left panels) and 2013 (right panels) including **(A,B)** the maximum photosynthetic rate (A_max_), **(C,D)** light saturation point (LSP), **(E,F)** light compensation point (LCP), **(G,H)** maximum apparent quantum yield (AQE), and **(I,J)** respiration. Inset figures show the average values across seasons with different letters indicating significant differences (*P* < 0.05) among the six treatments. Values represent mean ± SE (*n* = 3).

**Figure 4:**
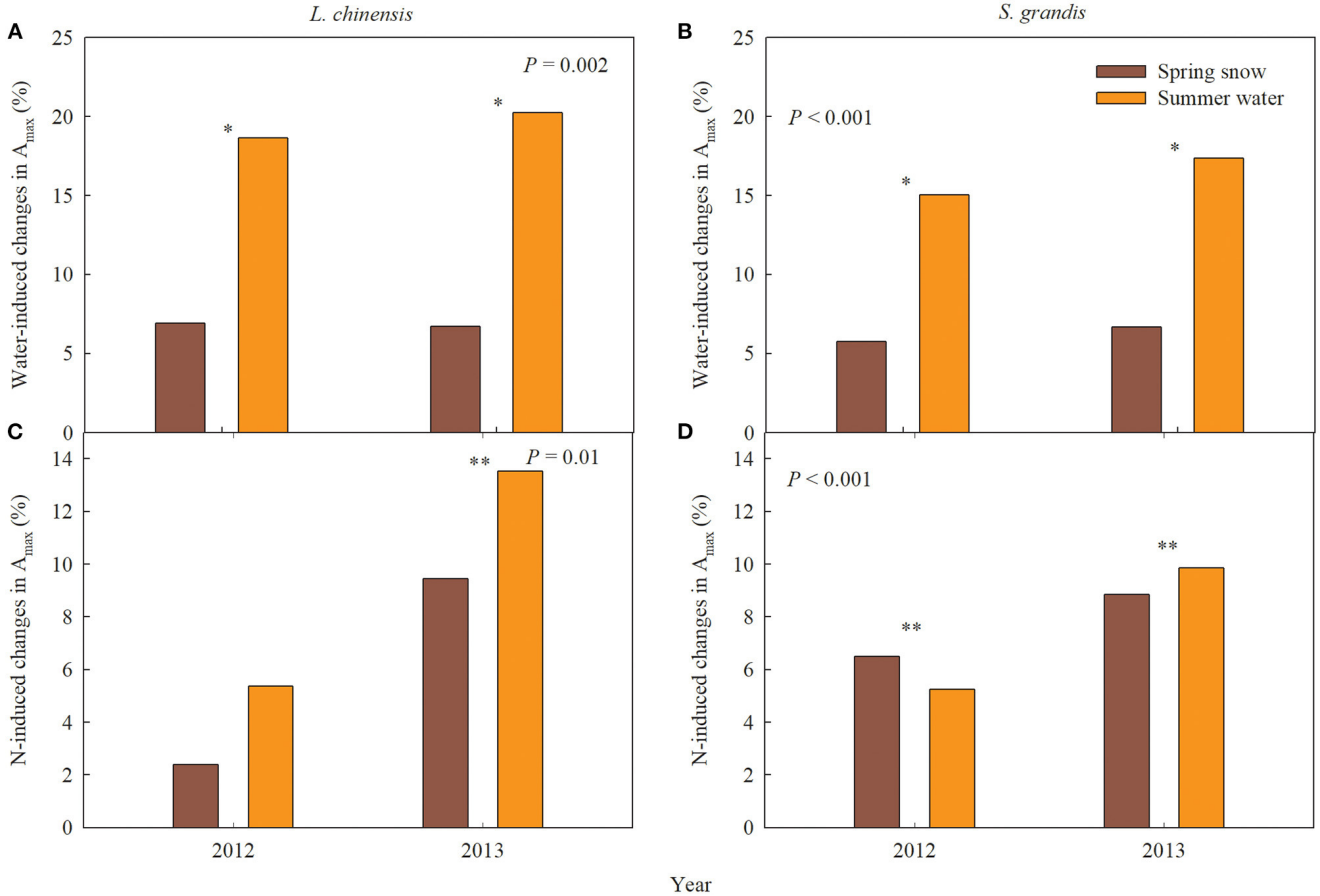
Water- and nitrogen (N)-induced changes in leaf carbon exchange of *Leymus chinensis* and *Stipa grandis* in 2012 and 2013, respectively. **(A,B)** Spring snow- and summer water-induced changes in the maximum photosynthetic rate (A_max_); **(C,D)** N-induced changes in the maximum photosynthetic rate (A_max_). *P*-values in the figures represent the difference between spring snow or summer water effects across 2 years. ** and * on each group represent significant differences in t-test, at *P* < 0.01 and *P* < 0.05 levels, respectively.

The effects of spring snow addition showed remarkable interannual variations in A_max_, light saturation point, light compensation point, the maximum apparent quantum yield, and respiration of the two dominant species between the two growing seasons (Tables 1, 2). Spring snow addition significantly increased A_max_ of *L. chinensis* by 6.9% and that of *S. grandis* by 5.8% in 2012 and light saturation point of *L. chinensis* in 2013, but the effect was insignificant on other variables in either year (Figures 2, 3, Tables 1, 2). The effects of summer water addition on A_max_ of *L. chinensis* and *S. grandis* were more pronounced than spring snow addition in both growing seasons (Figures 4A,B). Specifically, summer water addition enhanced A_max_ of *L. chinensis* by 18.7% in 2012 and 20.3% in 2013 and increased that of *S. grandis* by 15.1% in 2012 and by 17.4% in 2013 (Figures 2A,B, 3A,B, Tables 1, 2). Summer water addition also increased light saturation point of *L. chinensis* and *S. grandis* in both years (Figures 2C,D, 3C,D, Tables 1, 2). Summer water addition decreased light compensation point of *S. grandis*, but had insignificant effects on that of *L. chinensis* in both years. Besides, summer water addition increased the maximum apparent quantum yield of *L. chinensis*, but had no significant effects on respiration of both species in either year (Figures 2E–J, 3E–J, Tables 1, 2).

**Table 1:** Results (*F-* and *P*-values) of mixed-model analysis on the effects of water (W; including spring snow and summer water), N application (N), and their interactions on the maximum photosynthetic rate (A_max_), light saturation point (LSP), light compensation point (LCP), maximum apparent quantum yield (AQE), respiration, stomatal conductance (G_s_), and transpiration rate (Tr) of *Leymus chinensis* in 2012 and 2013.

**Water addition**	**Year**	**Treatment**	**df1**	**df2**	**A**_**max**_		**LSP**		**LCP**		**AQE**		**Respiration**		**G**_**s**_		**Tr**	
					***F***	***P***	***F***	***P***	***F***	***P***	***F***	***P***	***F***	***P***	***F***	***P***	***F***	***P***
Spring snow addition	2012	W	1	96	4.02	0.05	0.25	0.62	0.49	0.49	0.08	0.73	0.66	0.42	7.34	0.01	0.01	0.94
		N	1	96	0.45	0.51	1.69	0.20	1.82	0.18	1.34	0.25	0.04	0.84	0.03	0.86	0.06	0.80
		W*N	1	96	0.90	0.35	0.25	0.62	2.71	0.10	0.88	0.35	0.92	0.34	3.02	0.09	0.68	0.41
	2013	W	1	96	1.71	0.20	3.42	0.07	0.03	0.86	0.77	0.38	0.12	0.74	0.28	0.60	0.21	0.65
		N	1	96	1.95	0.17	0.23	0.64	1.83	0.18	0.86	0.36	2.70	0.10	0.004	0.95	1.52	0.22
		W*N	1	96	0.15	0.70	0.004	0.95	0.15	0.70	0.41	0.52	0.41	0.52	0.41	0.52	2.53	0.12
Summer water addition	2012	W	1	96	24.97	<0.001	5.29	0.02	0.003	0.95	3.93	0.05	1.61	0.21	12.18	0.001	2.00	0.16
		N	1	96	2.32	0.13	0.51	0.48	2.33	0.15	1.13	0.29	2.63	0.11	0.09	0.76	1.25	0.27
		W*N	1	96	0.002	0.96	0.01	0.93	3.12	0.08	1.53	0.22	5.30	0.02	1.16	0.29	0.01	0.93
	2013	W	1	96	12.78	0.001	9.91	0.002	1.26	0.27	0.16	0.69	0.01	0.93	6.23	0.01	6.47	0.01
		N	1	96	4.94	0.03	1.80	0.18	0.33	0.57	0.23	0.63	0.81	0.37	0.94	0.33	0.003	0.96
		W*N	1	96	0.25	0.62	0.91	0.34	1.58	0.21	1.14	0.29	1.92	0.17	0.38	0.54	0.21	0.65

*The degrees of numerator and denominator are represented by df1 and df2, respectively*.

**Table 2:** Results (*F-* and *P*-values) of mixed-model analysis on the effects of water (W; including spring snow and summer water), N application (N), and their interactions on the maximum photosynthetic rate (A_max_), light saturation point (LSP), light compensation point (LCP), maximum apparent quantum yield (AQE), respiration, stomatal conductance (G_s_), and transpiration rate (Tr) of *Stipa grandis* in 2012 and 2013.

**Water addition**	**Year**	**Treatment**	**df1**	**df2**	**A**_**max**_		**LSP**		**LCP**		**AQE**		**Respiration**		**G**_**s**_		**Tr**	
					***F***	***P***	***F***	***P***	***F***	***P***	***F***	***P***	***F***	***P***	***F***	***P***	***F***	***P***
Spring snow addition	2012	W	1	96	4.25	0.04	0.07	0.79	0.99	0.32	1.80	0.18	1.04	0.31	3.29	0.07	0.01	0.93
		N	1	96	5.33	0.02	8.23	0.01	0.58	0.45	0.87	0.35	0.14	0.71	1.51	0.22	0.36	0.55
		W*N	1	96	0.002	0.96	0.13	0.72	1.04	0.31	0.52	0.47	0.52	0.47	5.87	0.02	1.56	0.21
	2013	W	1	96	1.62	0.21	1.79	0.18	0.78	0.38	0.79	0.38	0.004	0.95	0.002	0.97	0.11	0.74
		N	1	96	4.35	0.04	1.89	0.17	0.51	0.48	0.02	0.90	1.03	0.31	0.09	0.77	1.64	0.20
		W*N	1	96	0.13	0.72	3.22	0.08	2.93	0.09	0.38	0.54	4.49	0.04	0.01	0.93	2.90	0.09
Summer water addition	2012	W	1	96	38.30	<0.001	3.14	0.08	3.00	0.09	0.09	0.76	0.14	0.71	14.81	<0.001	3.06	0.08
		N	1	96	5.17	0.03	7.14	0.01	0.43	0.52	0.10	0.75	0.97	0.33	0.08	0.78	1.55	0.22
		W*N	1	96	0.21	0.65	0.08	0.78	0.04	0.85	1.65	0.20	0.45	0.51	0.69	0.41	0.34	0.56
	2013	W	1	96	13.01	<0.001	6.81	0.01	3.86	0.05	0.27	0.61	0.89	0.35	12.10	<0.001	7.72	0.01
		N	1	96	4.24	0.04	2.37	0.13	2.20	0.14	0.13	0.72	1.70	0.20	0.89	0.35	0.54	0.46
		W*N	1	96	0.23	0.63	3.27	0.07	0.04	0.85	0.83	0.36	0.003	0.95	0.31	0.58	1.30	0.26

*The degrees of numerator and denominator are represented by df1 and df2, respectively*.

Applying N combined with spring snow addition increased the A_max_ of *S. grandis* by 6.5% in 2012 and by 8.8% in 2013 (Figure 4D). They also increased respiration of *L. chinensis* in 2013 and light saturation point of *S. grandis* in 2012, but had no significant effects on other photosynthetic parameters of the two species in both growing seasons (Figures 1, 2, Tables 1, 2). Applying N with summer water addition increased A_max_ of *L. chinensis* by 13.5% in 2013 and that of *S. grandis* by 5.2% in 2012 and by 9.9% in 2013. They also enhanced light saturation point of *S. grandis* in 2012 (Figures 2, 3, Tables 1, 2). The responses of A_max_ to N addition combined with summer water addition were stronger than with spring snow addition in 2013 for *L. chinensis*, but were weaker in 2012 for *S. grandis* (Figures 4C,D).

There were significant interactive effects between N addition and spring snow addition on respiration of *S. grandis* in 2013 and between N addition and summer water addition on respiration of *L. chinensis* in 2012.

### Leaf Physiological Traits

**Figure 5:**
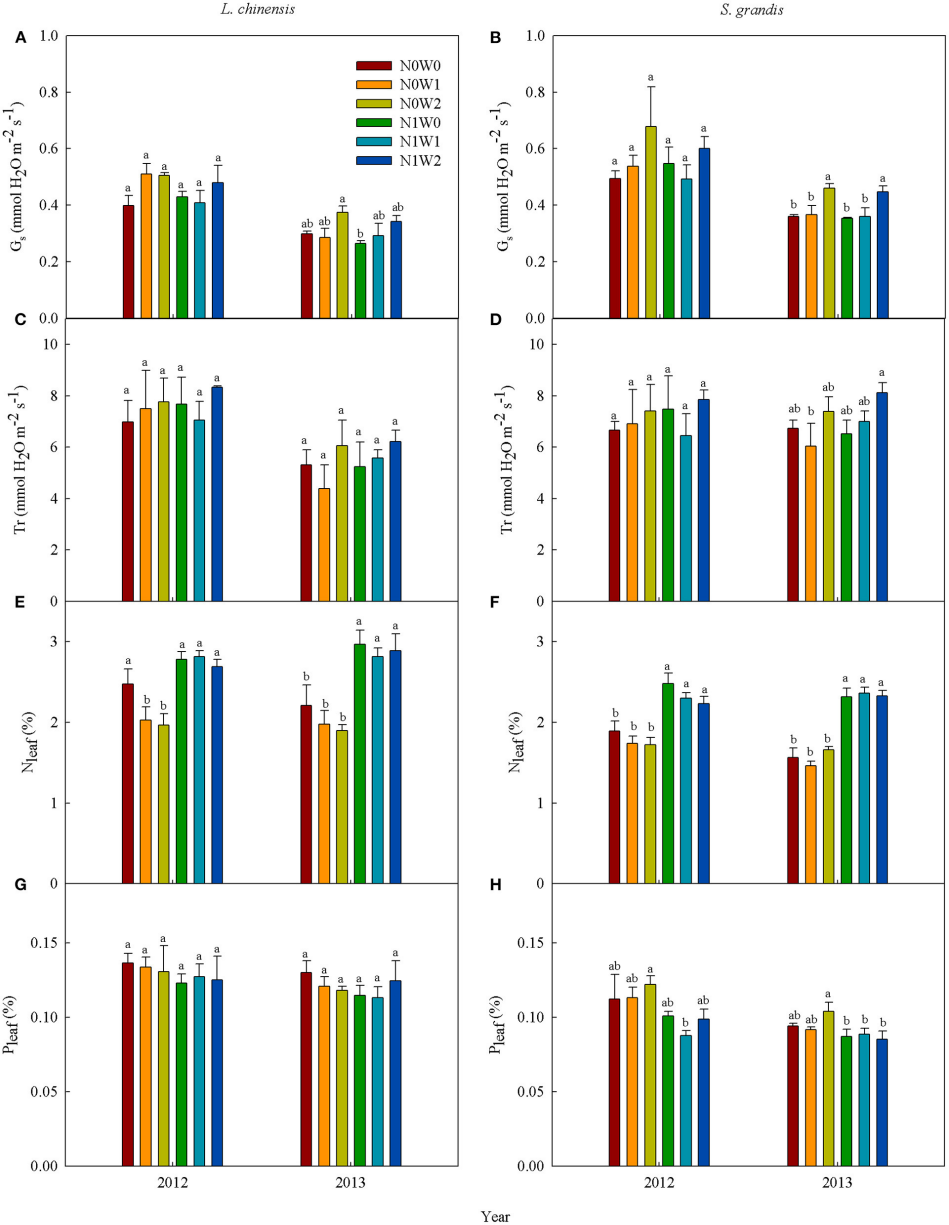
Mean values of leaf photosynthetic characteristics of *Leymus chinensis* (left panels) and *Stipa grandis* (right panels) across seasons in 2012 and 2013, respectively. **(A,B)** Stomatal conductance (G_s_), **(C,D)** transpiration rate (Tr), **(E,F)** leaf N concentration (N_leaf_), and **(G,H)** leaf P concentration (P_leaf_). Different letters indicate significant differences (*P* < 0.05) among six treatments. Values represent mean ± SE (*n* = 3).

Spring snow addition increased stomatal conductance of both species, but decreased P_leaf_ of *S. grandis* in 2012 and had no significant effects on other variables (Figures 5A–D, Tables 1, 2). Summer water addition increased stomatal conductance of both species, respectively, in 2012 and 2013 (Figures 5A,B). It also enhanced transpiration rate of *L. chinensis* in 2013 and that of *S. grandis* in 2012 and 2013 (Figures 5C,D, Tables 1, 2), but decreased N_leaf_ of *L. chinensis* in 2012 (Figures 5E,F, Table 3).

**Table 3:** Results (*F-* and *P*-values) of two-way ANOVA on the effects of water (W; including spring snow and summer water), N application (N), and their interactions on leaf N concentration (N_leaf_, %) and leaf P concentration (P_leaf_, %) of *Leymus chinensis* (N_*LC*_ and P_*LC*_) and *Stipa grandis* (N_*SG*_ and P_*SG*_) in 2012 and 2013.

**Water addition**	**Year**	**Treatment**	**df**	**N**_**LC**_		**N** ***_*****SG*****_***		**P** ***_*****LC*****_***		**P** ***_*****SG*****_***	
				***F***	***P***	***F***	***P***	***F***	***P***	***F***	***P***
Spring snow addition	2012	W	1	0.41	0.54	0.84	0.39	0.45	0.52	5.56	0.05
		N	1	21.13	0.002	12.32	0.01	2.45	0.16	8.00	0.02
		W*N	1	1.22	0.30	0.06	0.81	0.45	0.52	0.22	0.65
	2013	W	1	0.56	0.48	0.31	0.60	0.44	0.54	1.78	0.22
		N	1	6.43	0.04	67.86	<0.001	1.78	0.22	0.44	0.52
		W*N	1	0.14	0.72	0.38	0.56	1.00	0.35	0.00	1.00
Summer water addition	2012	W	1	9.19	0.02	0.81	0.40	1.80	0.22	0.19	0.31
		N	1	71.05	<0.001	10.85	0.01	45.00	<0.001	5.76	0.04
		W*N	1	6.00	0.04	0.13	0.73	24.20	0.001	0.05	0.83
	2013	W	1	0.24	0.64	0.06	0.82	0.08	0.79	0.56	0.48
		N	1	11.93	0.01	48.59	<0.001	0.31	0.59	1.56	0.25
		W*N	1	1.37	0.28	1.87	0.21	1.92	0.20	0.56	0.48

When combined with spring snow addition, the application of N did not affect stomatal conductance and transpiration rate of either species in either growing season (Figures 5A,B, Tables 1, 2). Besides, N addition significantly increased N_leaf_ of both species, respectively, in 2012 and 2013, but decreased P_leaf_ of *S. grandis* significantly in 2012 (Figures 5E–H, Table 3).

When combined with summer rainfall, the application of N had insignificant effects on stomatal conductance and transpiration rate of the two species over the two growing seasons (Tables 1, 2). Besides, it enhanced N_leaf_ of *L. chinensis* and *S. grandis* in both years, but decreased P_leaf_ of *L. chinensis* and *S. grandis* in 2012 (Figures 5E–H).

There were significant interactions between spring snow and N application on the stomatal conductance of *S. grandis* and between summer water addition and N application on N_leaf_ and P_leaf_ of *L. chinensis* in 2012.

### Effects of Abiotic Factors on A_max_

**Figure 6:**
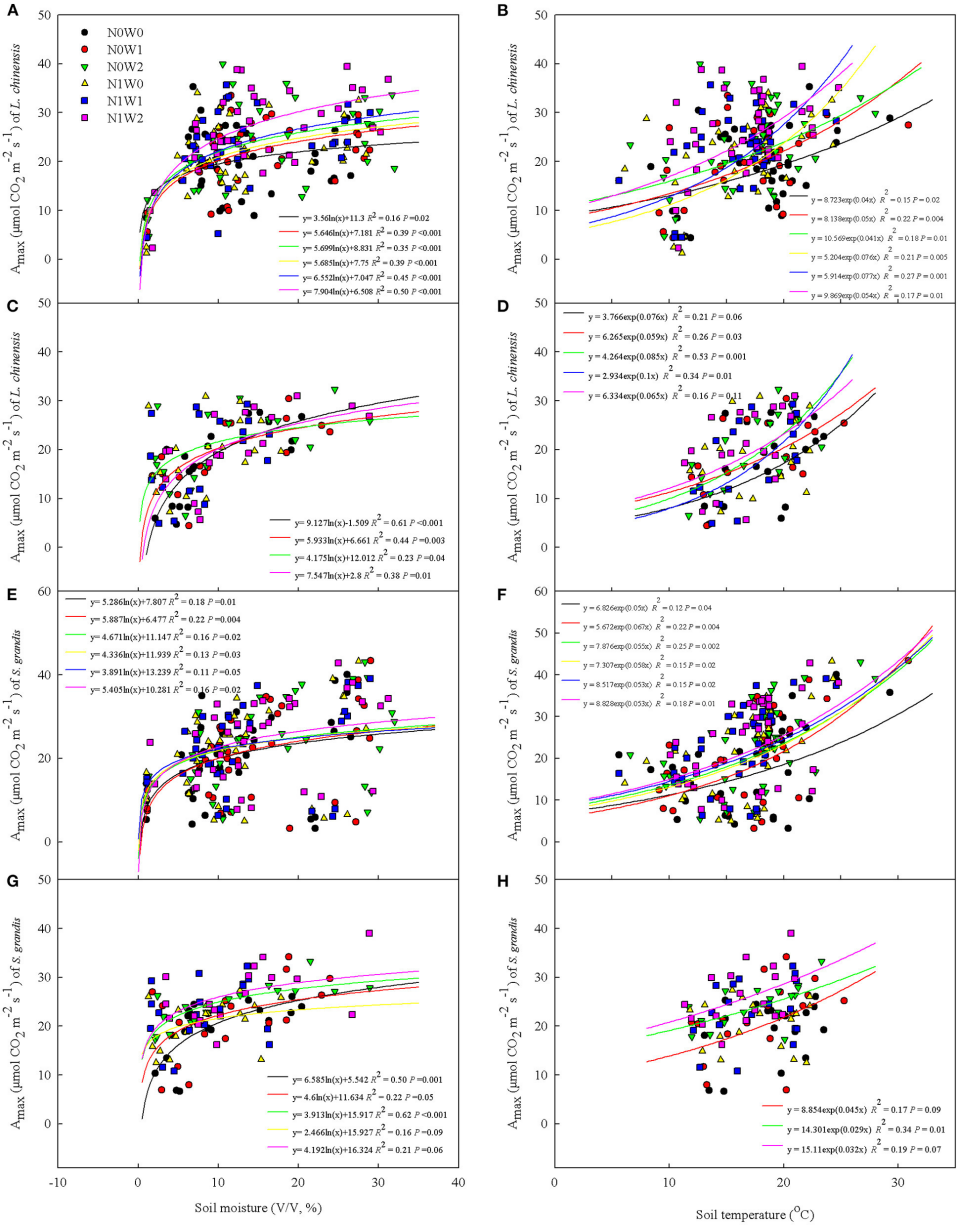
Relationships of the maximum photosynthetic rate (A_max_) with soil moisture (left panels) and soil temperature (right panels) of two dominant species across 2012 and 2013. **(A,B)** A_max_ of *Leymus chinensis* in the early growing season (from end-May to mid-August), **(C,D)** A_max_ of *Leymus chinensis* in the later growing season (from end-August to end-September), **(E,F)** A_max_ of *Stipa grandis* in the early growing season (from end-May to mid-August), and **(G,H)** A_max_ of *Stipa grandis* in the later growing season (from end-August to end-September).

The A_max_ of both species showed a logarithmic relationship with volumetric soil moisture in all treatments across 2012 and 2013, except N1W0 and N1W1 treatments for *L. chinensis* and N1W1 treatment for *S. grandis* in the later growing season (from end-August to end-September) (Figures 6A,C,E,G). The A_max_ of *L. chinensis* and *S. grandis* both showed an exponential growth relationship with soil temperature in all treatments across 2012 and 2013, except N1W0 treatment for *L. chinensis* and N0W0, N1W0, and N1W1 treatments for *S. grandis* in the later growing season (Figures 6B,D,F,H).

**Figure 7:**
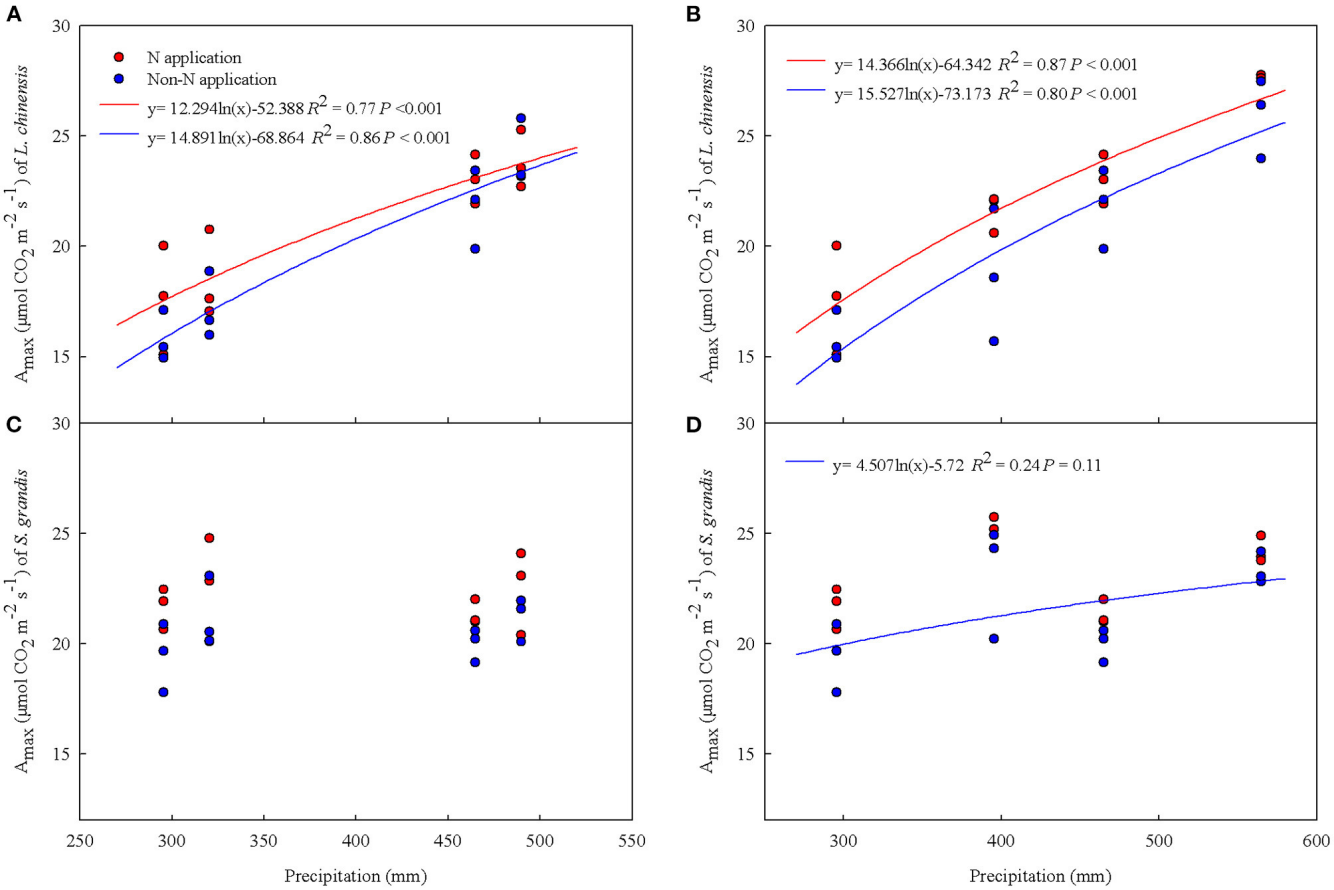
Relationships of the maximum photosynthetic rate (A_max_) of *Leymus chinensis* and *Stipa grandis* with precipitation (from previous November to October amount) under water addition treatment with N application and non-N application across 2012 and 2013. **(A)** A_max_ of *Leymus chinensis* under spring snow addition treatment, **(B)** A_max_ of *Leymus chinensis* under summer water addition treatment, **(C)** A_max_ of *Stipa grandis* under spring snow addition treatment, and **(D)** A_max_ of *Stipa grandis* under summer water addition treatment.

In addition, the A_max_ showed a significantly logarithmic relationship with increases in precipitation under spring snow and summer water addition treatments regardless of N application for *L. chinensis*, but only a marginally logarithmic relationship with precipitation for *S. grandis* under summer water addition with non-N application over the two growing seasons (Figure 7).

## Discussion

### Effects of Precipitation Increase on Leaf C Exchange

Leaf photosynthetic characteristics reflect physiological adaptations of plants to changes in a specific environmental condition. Because water is a key limiting factor for plant growth, especially in arid and semi-arid areas, changes in precipitation (in the form of either spring snowfall or summer rainfall) are expected to affect plant growth via changing soil water availability and leaf photosynthetic characteristics such as A_max_ and stomatal conductance (Nippert et al., 2009). Most previous studies have demonstrated positive effects of increasing water supply on leaf photosynthesis (Song et al., 2016; Ali et al., 2018; Hui et al., 2018), although inconsistent results, ranging from being weak to negative, are also reported (Hartman et al., 2012; Yu et al., 2015). Besides, previous studies have also shown that the magnitude of changes in leaf photosynthetic characteristics induced by precipitation alteration depends closely on the amount of background precipitation (Fay et al., 2011; Zheng et al., 2011).

In this study, the effects on leaf C exchange varied remarkably during growing seasons with the form of the additional water either as spring snow or as summer water. Our results showed that the effect of increasing water on leaf photosynthetic characteristics was generally weaker for spring snow increase than for summer water increase. The most probable reason is that snowfall takes only a small part of the total annual precipitation in the study area. Besides, it occurs generally in the non-growing seasons, which mismatch the time of plant growth. A previous study showed that grassland was increasingly vulnerable to direct effects of climate extremes through affecting ecosystem function and phenology as changes in key traits of plant species (Knapp et al., 2020). We found that increases in spring snowfall and summer rainfall could significantly increase the capacity of both species' C assimilation by increasing light saturation point, stomatal conductance, and transpiration rate while decreasing light compensation point. Therefore, our findings highlight the importance that responses of both the species to future precipitation increase can be reflected via changes in key photosynthetic properties, which may finally affect ecosystem functioning and phenology.

In addition, previous studies showed that predicting responses of leaf C exchange and subsequent responses of semi-arid ecosystems C budget to precipitation required knowledge of timing of precipitation change (Craine et al., 2012; Post and Knapp, 2020). In this study, our results also showed that impacts of spring snow and summer water addition on leaf C exchange of the two dominant species varied among different years. The effects of spring snow addition were greater in 2012 when the background snowfall was around the long-term average value of the study area, but weaker in 2013 when the background snowfall was far greater than its average level. Similarly, the effects of summer water addition were weaker in 2012 when the background precipitation was higher than the long-term average of the study area, but stronger in 2013 when the background precipitation was at its average level. These results suggest that the enhancive effects of precipitation increase on leaf C exchange of the two dominant species depend highly on the amount of background precipitation with the effect that may be weakened when the background precipitation is high.

According to the results from a previous study, precipitation events affect ecological processes by soil moisture pulses in arid and semi-arid ecosystems (Post and Knapp, 2020), with soil moisture being able to explain the variation of leaf C assimilation as high as 52% (Fay et al., 2011). Our results also showed that there was a significantly positive relationship between A_max_ and soil moisture, which indicates that improvement in water supply on soil moisture could be an important underlying mechanism for the enhancement of the capacity of plant C assimilation by precipitation increase.

### Effects of Additional N on Leaf C Exchange

Nitrogen supply affects plant growth and productivity, but the effect is more complex than expected and the mechanism on leaf photosynthetic characteristics is in fact not fully understood. Our results showed that N application along with spring snow addition increased the leaf C exchange capacity (A_max_) of *S. grandis* and along with summer rainfall addition treatment significantly increased the leaf C exchange capacity of both species except the case for *L. chinensis* in 2012. The increasing effect of N addition might be a resultant of increasing light saturation point and N_leaf_, but decreasing P_leaf_ as well, all of which were important physiological indicators of a plant's potential photosynthetic capacity (Luo et al., 2019). This also suggests that leaf C exchange of both the studied species is N-dependent and the N effect can be regulated by the water supply amount. A previous study showed that N application promoted phosphatase activity leading to P recycling stronger (Schleuss et al., 2020), which suggests that N addition aggravates the response of plant to P resulting in weakened response of leaf C exchange to N addition at our study site.

The significantly positive relationship between A_max_ and soil temperature partially explained the effect of N application on the capacity of plant C assimilation (Figure 6). With an increase in soil temperature and perhaps soil moisture as well, A_max_ was greater in N application than in non-N application plots for both species in the early growing season, but not later. These results suggest that N availability regulated water effects in the responses of plant performance mainly in the early growing season. In addition, the increasing effects of N application on leaf C exchange of the two species also changed when background precipitation varied (Figures 4C,D), indicating that the natural precipitation also adjusted the effect of N application, which might lead to limitation of other resources such as the availability of P in this semi-arid area.

### Different Responses of Leaf C Exchange to Water and N Application

Different response patterns of the photosynthetic properties between the two dominant species under precipitation increase and N application can provide insights into the effects of climate changes on the structure of plant communities. Previous studies have shown that precipitation changes will significantly affect the plant photosynthesis and leaf level C exchange, which was corroborated in community-level CO_2_ exchange (Zhang R. et al., 2017), resulting in the variation of future population and community structure depending on the different magnitude of responses among the community compositional species (Silletti and Knapp, 2001; Nippert et al., 2009). In this study, although the patterns of light responding properties of the two species were similar to changes in spring snowfall and summer rainfall, the A_max_ of *L. chinensis* had an obviously higher increasing magnitude than that of *S. grandis* in both study years (Figures 4A,B), suggesting that the response of *L. chinensis* was stronger than that of *S. grandis* to change in precipitation in the study area. This may be because the relationships of A_max_ with soil moisture were more tightly related for *L. chinensis* than for *S. grandis* in both the early and later growing season. In addition, the A_max_ of *L. chinensis* was more tightly related with an increase in precipitation than that of *S. grandis*, suggesting that it is more conducive to the development of *L. chinensis* under the scenario of increasing precipitation in future. Moreover, responses of the A_max_ to N application were also greater for *L. chinensis* than for *S. grandis* under summer water addition treatment although only in 2013, while it was adverse under spring snow addition, suggesting that *L. chinensis* responses to N addition were more dependent on precipitation than *S. grandis*. The difference in the magnitude of responses between the two species may also result from their different adaptation strategies in terms of their rooting systems. *S. grandis* generally had fibrous rooting system distributing in relatively shallow soils, while *L. chinensis* showed strong rhizomatous rooting system which could distribute deeper in the soil profile (Chen et al., 2001; Zhang et al., 2021). Therefore, *L. chinensis* could be more beneficial than *S. grandis* for absorbing water and N resources from deeper soils, especially under the summer water addition. This corroborates results from previous studies which indicated that habitats for distribution of *L. chinensis* were generally wetter and more fertile than those of *S. grandis* (Chen et al., 2005).

Our results implicate that *L. chinensis* and *S. grandis* will respond differently to water and N availability increase due to their different root morphology and resource use strategy which corroborates the results of our earlier studies (Hasi et al., 2021; Zhang et al., 2021). Our previous work showed that the abundance and aboveground biomass of *L. chinensis* increased significantly under water addition with or without N application (Zhang et al., 2021), and this study suggests that physiological responses of that species may mirror those higher-order ecosystem responses. Therefore, different responses of two dominant species may lead to changes in the ecosystem structure and functioning when facing global change factors including both increasing precipitation and enhancive N deposition in this study area.

However, the sample size is relatively small in this study to fully explain the influencing mechanism of spring snow or summer water addition and N application on two dominant species leaf C exchange, although we tried to increase the monitoring frequency as much as possible. Therefore, more studies are needed to further explain the potential mechanism on the responses of plant performance to precipitation increase and N deposition in future.

## Conclusion

Our results showed that summer water addition effects on leaf C exchange of *L. chinensis* and *S. grandis* were greater than spring snow addition. Correspondingly, N effects were more pronounced when combined with summer rainfall than spring snow addition by changing their key photo-physiological traits reflecting different adaptation strategies. This explains a physiological-based mechanism for potential transition in plant species composition as *L. chinensis* was more sensitive than *S. grandis* under water and N application. Therefore, differentiated responses of plant species in resource use and C assimilation may result in changes in community structure and further affect ecosystem functioning when facing global change scenarios such as increases in precipitation and N availability.

## Data Availability Statement

The raw data supporting the conclusions of this article will be made available by the authors, without undue reservation.

## Author Contributions

XZ conducted this study, made measurements, and wrote the draft of manuscript. PZ helped in the data analysis. JH initiated the experimental design and made final manuscript revision. All authors contributed to the article and approved the submitted version.

## Funding

This study was financially supported by the National Natural Science Foundation of China (31800381 and 32071562), the Scientific and Technological Innovation Programs of Higher Education Institutions in Shanxi (2019L0366), the Research Project for Outstanding Doctor Work Award Fund in Shanxi Province (SXYBKY201746), the Science and Technology Innovation Fund of Shanxi Agriculture University (2017YJ11), and the Shanxi Province Graduate Education Innovation Project (2020BY054).

## Conflict of Interest

The authors declare that the research was conducted in the absence of any commercial or financial relationships that could be construed as a potential conflict of interest.

## Publisher's Note

All claims expressed in this article are solely those of the authors and do not necessarily represent those of their affiliated organizations, or those of the publisher, the editors and the reviewers. Any product that may be evaluated in this article, or claim that may be made by its manufacturer, is not guaranteed or endorsed by the publisher.
